# Short-term forecasting of solar irradiance using decision tree-based models and non-parametric quantile regression

**DOI:** 10.1371/journal.pone.0312814

**Published:** 2024-12-11

**Authors:** Amon Masache, Precious Mdlongwa, Daniel Maposa, Caston Sigauke

**Affiliations:** 1 Department of Statistics and Operations Research, National University of Science and Technology, Bulawayo, Zimbabwe; 2 Department of Statistics and Operations Research, University of Limpopo, Sovenga, South Africa; 3 Department of Mathematical and Computational Sciences, University of Venda, Thohoyandou, South Africa; Aalto University, FINLAND

## Abstract

The renewable energy industry requires accurate forecasts of intermittent solar irradiance (SI) to effectively manage solar power generation and supply. Introducing the random forests (RFs) model and its hybridisation with quantile regression modelling, the quantile regression random forest (QRRF), can help improve the forecasts’ accuracy. This paper assesses the RFs and QRRF models against the quantile generalised additive model (QGAM) by evaluating their forecast performances. A simulation study of multivariate data-generating processes was carried out to compare the forecasting accuracy of the models when predicting global horizontal solar irradiance. The QRRF and QGAM are completely new forecasting frameworks for SI studies, to the best of our knowledge. Simulation results suggested that the introduced QRRF compared well with the QGAM when predicting the forecast distribution. However, the evaluations of the pinball loss scores and mean absolute scaled errors demonstrated a clear superiority of the QGAM. Similar results were obtained in an application to real-life data. Therefore, we recommend that the QGAM be preferred ahead of decision tree-based models when predicting solar irradiance. However, the QRRF model can be used alternatively to predict the forecast distribution. Both the QGAM and QRRF modelling frameworks went beyond representing forecast uncertainty of SI as probability distributions around a prediction interval to give complete information through the estimation of quantiles. Most SI studies conducted are residual and/or non-parametric modelling that are limited to represent information about the conditional mean distribution. Extensions of the QRRF and QGAM frameworks can be made to model other renewable sources of energy that have meteorological characteristics similar to solar irradiance.

## 1 Introduction

Photovoltaic power generation directly and heavily depends on solar irradiance (SI). The rapid fluctuating uncertainty characteristics of SI make photovoltaic power generation have intermittent and uncontrollable characteristics that greatly impact the stability of solar power systems [1]. SI data is often characterised by significant covariate multicollinearity, existence of outliers, heavy right-tailed, platykurtic, lot of noise and no known probability distribution [2–5]. SI data sets are among complex data sets and include meteorological covariates that are also characterised by rapidly fluctuating uncertainties. The relationship structures between SI and its covariates are also unknown. Quantile regression (QR) is robust and gives sparse solutions in such data situations. The literature has proven that non-parametric QR approaches outperform the prediction accuracy of parametric QR [2, 3]. In addition to limited SI modelling studies in Southern Africa, there is a lack of comparative investigation of non-parametric QR-based models when predicting SI data. There is a need to investigate the non-parametric QR-based models to study how the predictive frameworks can be applied to improve the forecasting accuracy of SI. Non-parametric QR predictive models are new to SI modelling. Studies that have applied non-parametric QR models to forecast SI data from Southern Africa so far were done by [3] to use a partially linear additive quantile regression (PLAQR), and [2] to apply an additive quantile regression (AQR) model. Recently [6] concluded that the new quantile generalised additive model (QGAM) forecasted SI data was nearly the best among the known non-parametric QR frameworks that model additive effects. Another new non-parametric QR which has not been considered for SI forecasting is the quantile regression random forest (QRRF) model, to the best of our knowledge. Introducing the QRRF model to SI studies adds more information that potentially enhances the forecasting accuracy. Therefore, this paper seeks to provide a proof-of-concept for the use of the QRRF model in the study of SI forecasting. The QRRF is a hybrid model of QR and a Random forests (RFs) model and the RFs model is a decision tree-based model. There are decision trees in RFs models that can learn highly irregular patterns in data with low bias [7]. The performances of these decision trees are improved through the ensembling algorithm [8]. In addition, the RFs model has a reputation for good predictive performance when using many covariates with non-linear relationships [9, 10]. That has made the RFs model a popular technique for regression and classification with complex data sets [11, 12].

### 1.1 Review of related literature

To the best of our knowledge, no study has been conducted to model SI data from Southern Africa using RFs. However, a notable number of studies from other regions can be cited. In most European studies, RFs have been found to perform better than residual modelling frameworks like Support Vector Machine (SVM) and multiple linear regression (MLR) models. Though [13] found Artificial Neural Networks (ANNs) to outperform RFs, the RF model was, in turn, better than MLR and SVM when modelling Galicia (Spain) SI data. In contrast, earlier studies from [8], who used SI data from France and [14], who modelled Gorakhpur (India) SI data, found RF modelling to outperform the ANN model. The other models considered by the two separate studies were smart persistent, multivariate adaptive regression splines (MARS), classification and regression tree (CART) and M5 models. An M5 model is based on a binary decision tree having linear regression functions at the leaf nodes. When [15] used eight different locational data sets from India, RFs also performed better than ANNs. However, the extreme gradient boost (XGB) model had the minimum root mean square error (RMSE) among the three models compared. Due to the different data qualities, open weather services do not guarantee the highest quality of forecasted data, and confusing contradictions may occur. However, [6] concluded that machine learning models perform differently in different locational data sets.

The above review of RF application-related literature shows that the study from [13] is one of the few studies where FRs were outperformed by ANNs. Though [16] concluded that single site results cannot be generalised to other different sites from different regions, we can safely perceive that comparison performance results between RFs and ANNs will be the same when using Southern Africa SI data. Ref. [16] had classified SI data from Ajaccio (Italy), Tilos (Turkey) and Odeillo (France) as low, medium and high meteorological variability data sets, respectively. RFs and bagged regression trees (ensemble models) were the best among the eleven methods on Odeillo data sets. They attributed the performance to the additional layer of randomness to bagging created by RFs, giving them the robustness property and the ability to decrease overtraining risks. In another European study by [17] but with no meteorological covariates, RF modelling was superior to linear regression, K-nearest neighbourhood (KNN), SVM and extreme learning machine ensembles. Roof tilt, roof aspect and three mean roof horizon heights were inputs to model Swiss’s Romandie solar irradiation data. In Australia [12] found RFs to be good intrinsic model function selectors of their proposed multivariate empirical model decomposition coupled with ant colony optimisation and RF modelling framework. Ref [10] agrees with [9] that RF modelling has a reputation for good predictive performance when forecasting a response variable with non-linear and even unknown relationship structures with many covariates. The relationship structure of SI with many covariates is not yet known. As a result, we concur with [10, 18] that with RFs, prediction intervals can be constructed without dependence on any assumed link between covariates and the response. Therefore, the application of RFs to SI data modelling can help establish accurate solar power forecasts in the Southern Africa region.

However, it has to be noted that the RF model is limited in forecasting the conditional mean. In addressing this weakness [19] hybridised the RFs algorithm with quantile regression (QR) and developed the quantile regression forest (QRF), which can forecast the conditional distribution of the response through quantiles. Though [20] called the hybrid model a quantile regression random forest (QRRF), they found the algorithm to have a great ability to learn the response pattern with the provided training data. It provided uncertainty related to the internal functionality and chosen settings of the RFs algorithm. As a result, QRRF considers uncertainty that presents some aspects of model uncertainty. Another quite recent study was done by [21] on modelling precipitation. Their error decomposition confirmed that the QRF model improved on various deficiencies of the Integrated Multi-satellite retrievals Early Run for Global Precipitation Measurement Mission (IMERG-E) product. The ensemble assessment demonstrated that the quantile outputs provide reliable prediction spread and sharp prediction intervals. Their QRF model could do both the deterministic mean correction and probabilistic calibration. This means that the QRF model could forecast beyond the conditional mean. A conditional distribution forecast could be established. That is, reliable probabilistic information was provided from the ensemble outputs with different dynamic predictor solutions. Ref. [22] also showed that the QRF model could be applied to correction of skewed distribution for a meteorological feature similar to precipitation. Forecasts were improved, and the performance gain was large in all situations by using other meteorological features as covariates for the post-processing snow height. Other meteorological studies by [23, 24] demonstrated that QRF models provided sharp and reliable probability forecasts than ensemble model outputs (EMOS). Their results indicated that QRF does not constrain the output probability density function, and the improvement was more consequent for non-Gaussian features like surface temperature and wind speed.

In contrast, when [25] compared the QRF model with other nine models, including neural networks (NNs) for distributional regression post-processing of surface temperature, QRF outperformed by NNs. Unlike other meteorological features modelling studies reviewed here, embeddings to add station information were used in the global model. They also estimated network model parameters differently by optimising the continuous ranked probability score (CRPS). NNs outperformed the QRF model because its predicted quantiles are restricted to the range of the observed values. It then becomes more interesting when we consider the study by [26], who also called the hybrid model a QRRF algorithm. The authors used results from three short-term load point forecasting models based on multi-model deep NNs as inputs to a QRRF model to quantify the uncertainty of power load. The NN models were used to extract nultimodal spatial-temporal features containing more hidden information, and the proposed QRRF model outperformed the conventional quantile gradient-enhanced regression tree.

As an improvement to QRF modelling, [27] suggested adding a physical model because it provided more information about their next-day icing-related wind power production losses. Like in the study of [25], information from stations considered improved the QRF model. The models were also trained separately on different specific stations, though the resulting probabilistic forecast skill varied slightly between the station separating options. As a result, the authors recommended deciding on a data separation option. In other meteorological feature modelling studies, the QRF model also compared very well with the state-of-the-art kriging method on rainfall erosivity data modelling in Greece [28]. In soil, science [29] overcome the challenge of coupling between vegetation and surface scattering by proposing a QRF modelling in vegetation-covered areas used to estimate surface soil moisture (SSM). They used varying land polarimetric parameters, quantified the importance of the parameters and predicted the uncertainty intervals. Model performance was evaluated and compared well against data from the soil moisture active passive validation experiment 2012 and also against the copula quantile regression. However, the model could not accurately capture the peaks and valleys of SSM. In the same field of soil science but on Digital Soil Mapping (DSM) techniques, QRF modelling did not also perform quite well when [30] were addressing the reliance of DSM practitioners on machine learning (ML) techniques. When integrating the ML techniques with QR as a solution, they included an evaluation of QRF models that overestimated the uncertainty in all scenarios studied. In contrast, when [31] applied QRF as an extended ensemble method to model soil properties, the state-of-the-art and common geostatistical regression kriging method was outperformed. Later, [32] found the QRF model to exhibit outstanding results in predicting soil organic matter near the shore of Lake Orestiada in northern Greece as compared to RFs and geostatistical methods. QRF modelling was also found to address weaknesses in geostatistical methods when [33] studied coal properties and [34] investigated Nitrogen Oxide pollution in urban locations.

The QRF model is also a powerful modelling tool in many other fields of study like surgery [35, 36], banking [37–40], farming [41, 42], marine studies [43, 44] and pedestrian trajectories [45], to mention a few. However, with all these excellent performances in its application, the mean prediction of several trained and ensembled QRFs was used in the forecast verification and test whenever a model was fitted. Each time a training is repeated, the prediction accuracy is slightly different.

### 1.2 Rationale and contribution of the study

The main contribution of this study is that it compares decision tree-based models and quantile generalised additive modelling (QGAM) in predicting global horizontal irradiance (GHI). The QGAM was benchmark compared with other non-parametric QR-based additive effects models in our recent study [6] and the QGAM was found to be the near best among the additive models. Decision tree-based models guarantee that SI predictions can never be negative because RF predictions can never be below the smallest and above the largest values of the training set. That is, forecasts in RFs are bounded between the smallest and largest observed response values [10]. Like the QGAM, no parametric assumption is needed, and tree-based models are easily applicable to multi-predictor settings. Since there is still randomisation of covariate subsets within the QR hybridised RFs model, we propose to call the framework the quantile regression random forest (QRRF) model as discussed by [20, 26]. Through hybridisation with QR, we further estimated the prediction interval $\left[Q_{\tau_{1}}\left(x\right),Q_{\tau_{2}}\left(x\right)\right]$ of the response with a given probability $P(Q_{\tau_{1}}\left(x\right)<Y<Q_{\tau_{2}}\left(x\right)|X=x)=\tau$ where *τ* ∈ (0, 1) and $P\left[Y<Q_{\tau_{i}}\left(x\right)|X=x\right)=\tau_{i}$. Notwithstanding the powerful ability of the RFs model to learn irregular patterns in noisy data like SI with low bias, QRRF introduces the application of QR on an ensemble of decision trees. QRRF is a state-of-the-art non-parametric QR modelling that has not yet been applied to solar irradiance modelling to our knowledge. The modelling framework considers each combination of all covariates and uses an out-of-bag (OOB) sub-sample training data set when growing an ensemble of trees. The model randomly selects covariate subsets at each leaf node on split point selections and then combines weighted histograms in each tree from those target observations. QRRFs extend the modelling of relationships between the response and covariates by obtaining the conditional distribution after preserving all observed predicted values in the leaf node.

The inclusion of decision tree-based models in prediction accuracy studies of SI informs forecasting practitioners in the renewable energy industry and the body of knowledge in general about the new state-of-the-art modelling framework that has powerful abilities to learn irregular patterns and high uncertainties in data. Thus, unearthing modelling frameworks that can be used to improve the forecasting accuracy of SI. The modelling frameworks also involve computing variable importance values, a qualitative measurement of covariate effects. The study helps establish accurate forecasts of solar irradiation, improving the stability of solar power generation and effective management of renewable resources. Having accurate predictions can significantly impact decision-making processes and ultimately determine the success or failure of an endeavour.

### 1.3 Research highlights

In the current study, before the lasso via hierarchical pairwise interactions variable selection method was applied, lag1 and lag2 of global horizontal irradiance (GHI) were included in the data to model the nonlinear trend in SI. The study used covariates to SI recommended by [46]. The RFs framework was applied as a benchmark model, and the QRRF was fitted using the “quantregForest” package developed by [47]. A simulation study was conducted on the comparative investigation between the RFs, QRRF and QGAM frameworks. Cross-validations and out-of-sample diagnostic evaluations were done. Simulation results indicated that the QGAM framework outperformed the decision tree-based models in predicting GHI. The predictive models were applied to real-life data, and similar results were obtained. The QRRF model was found to have better prediction accuracy than the RF model. This meant hybridising the RFs model with QR improved the forecasting performance of the decision tree-based model.

## 2 Methodology

### 2.1 Decision tree-based models

The QRRF model is a RFs model hybridised with QR in such way that QR is applied to the RFs algorithm. That is, apart from what the RFs model does to keep only the mean of observations on each leaf node the QRRF further keeps the value of all observations and assesses the conditional distribution of the response variable [19]. The RFs model predicts the conditional mean which can over- or under-estimate extreme values in data samples. Hence, the QRRF model which estimates conditional quantiles that are not affected by the existence of outliers like in SI data, can be preferred to the RFs model. However, when running a QRRF model all principles of the RFs modelling are followed through and parameter tuning is done within the RFs algorithm.

#### 2.1.1 The RFs algorithm

**Fig 1:**
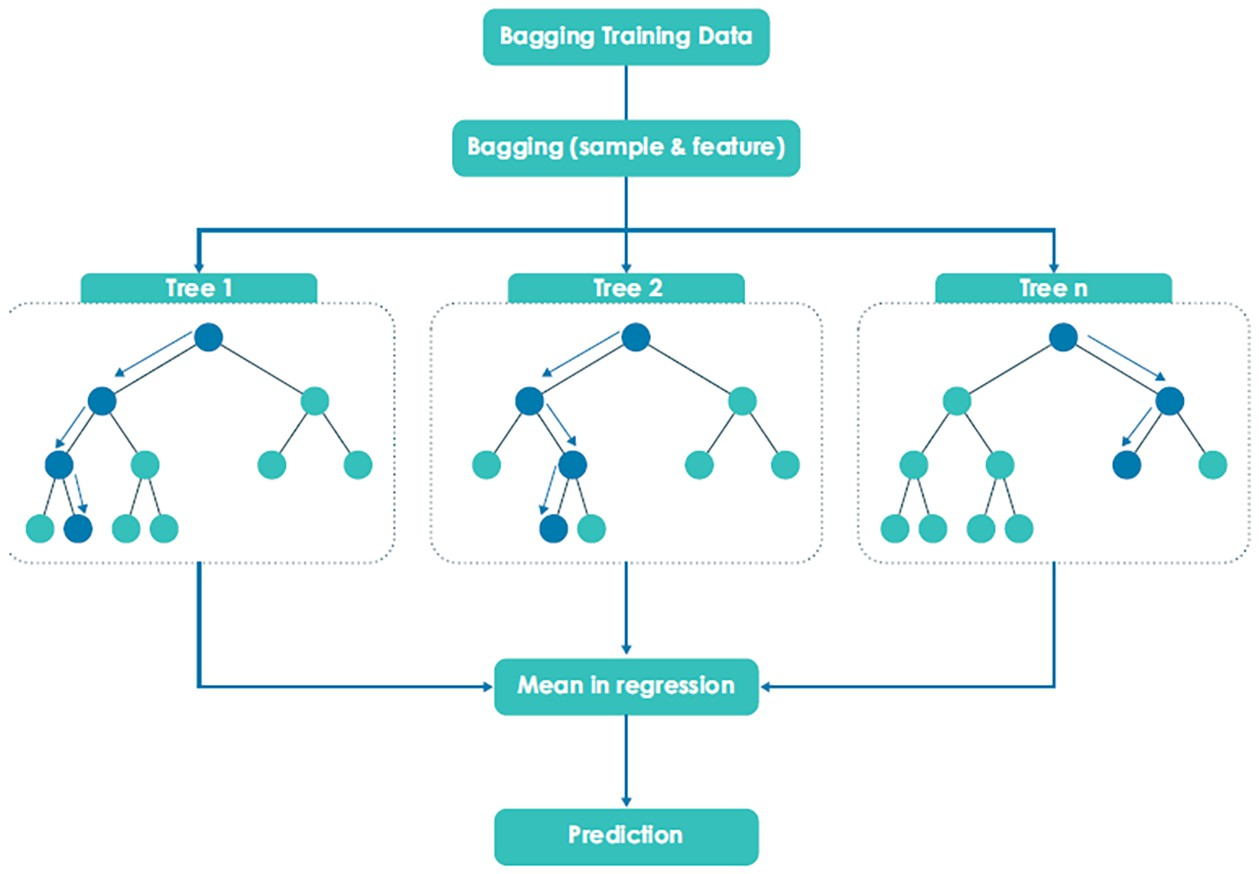
RFs regression tree.

The RFs algorithm is a class of non-parametric models, an ensemble of unbiased decision trees. Modelling is done through a system of classification performed by voting of a multiple of decorrelated decision trees called a forest. It is an improved model of the bagging regression tree [8]. The trees are independently developed on different subsets of attributes taken from a training set. These subsets are bootstrap sample bags, each a result of the random replacement selection of the same objects as the original set. The number of trees is the first parameter to choose and is set to the number of bootstrap samples that are randomly formed from the learning data set. However, [7] suggested a default of 500 trees. To reduce variance, trees trained on different parts of the same training set are averaged. As a result, bias increases and the model may lose interpretability. However, the performance of the final model is significantly boosted. The original dataset is extended by adding the so-called shadow features whose values are randomly permuted among the training cases to remove their correlations with a decision variable. RFs improve learning performance with a voting system given a set number of decision trees. That is, as new objects come in, all decision trees in the forest classify them and the final decision on the new objects is made through this voting system. Decision trees vote for the classification of objects which were not involved in their classification. The votes for a correct class are recorded for each tree, and the values of variables are randomly permuted across objects, and the classification is repeated. In summary, [7] highlighted that the RFs model exhibits characteristics of random feature selection, bootstrap sampling, OOB error estimation and full-depth decision tree growing. At first, the RFs model extracts some of the samples by bootstrap sampling and then randomly selects the features of these samples, as shown in Fig 1.

These two steps of random sampling make the RF model more tolerant to the noise in the data series and reduce the possibility of overfitting. However, the response range on RF modelling is limited by the domain of training features [27]. Each tree keeps only the mean of the observations in the leaf nodes and at each leaf node, a binary rule is created through the random selection of the number of splits. The second parameter to tune in RFs modelling is the number of leaves (variables to try) per tree grown. It is the best number of splits in each tree. This best number of splits is selected through a comparison of the estimated mean square error (MSE) in each split and with the obtained OOB data [48]. Introducing non-parametric QR to the RF model can keep more information in each leaf node. In addition to the mean, the QRRF model stores all observations’ values and further assesses the conditional distribution of the response. Thus, percentiles can now be calculated, and if the observations are unequally weighted, then the full conditional distribution of the response can be estimated [31]. This computation can be done accurately and efficiently even if the normality assumption to the conditional distribution does not hold. Therefore, the QRRF model inherits all the advantages of the RFs model.

#### 2.1.2 The QRRF modelling

**Fig 2:**
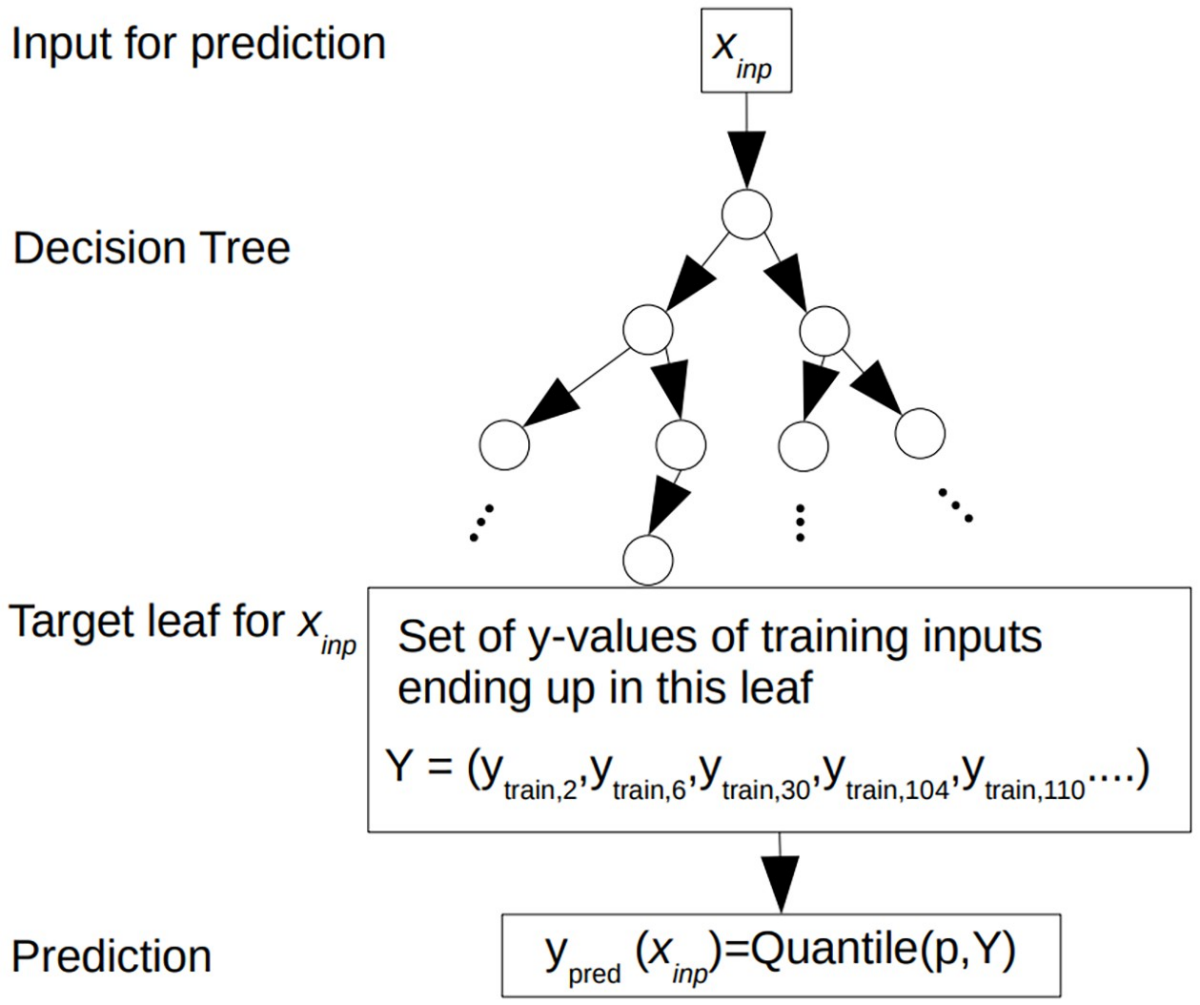
Illustration of a prediction from a trained single QRRF tree. Samples 2, 6, 30, 104 and 110 are training samples that would end up in a leaf node. The desired quantile is the prediction value required: *Source* [27].

A single decision tree is grown and used to predict, as shown in Fig 2.

Each tree is parsed separately, and then a weighted quantile of all the response values in the chosen leaf nodes is computed. This chosen leaf node contains a set of the response prediction values of all OOB training samples. All leaves in a single tree keep the raw distribution of the response variable. If the observations *y*_*i*_ are unequally weighted, then a good approximation of the full conditional distribution from the covariates *x*_*i*_;

$$\begin{array}{c} F\left(y|x=x_{i}\right)=P\left(y\leq y_{i}|x=x_{i}\right)=E\left[\text{I}\left(y_{i}\leq y\right)\right], \end{array}$$ (1)

can be delivered as in Fig 2. We can define an approximation to the expectation by the positive weighted mean over the observations of *I*(*y*_*i*_ ≤ *y*) as

$$\begin{array}{c} \hat{F}\left(y|x=x_{i}\right)=\sum_{i=1}^{n}w_{i}\left(x\right)\text{I}\left(y_{i}\leq y\right), \end{array}$$ (2)

where $w_{i}\left(x\right)=\frac{1}{k}\sum_{t=1}^{k}w_{i}\left(x,\theta_{t}\right)$. We note that *E*[*Y*|*X* = *x*] is approximated by the averaged prediction of *k* single decision trees. Each single tree is constructed with an iid vector *θ*_*t*_ (*t* = 1, 2, …, *k*), which indicates the *t*^*th*^ tree for any new observation. That is, for any *τ*-quantile level defined by

$$\begin{array}{c} \tau =P(y<Q_{\tau }\left(x\right)|x=x_{i}),\;\;\;\;\tau \in \left(0,1\right) \end{array}$$ (3)

the *τ*-quantile of *y* is given by the infimum over *q* = (00, 01, …, 99) for which *P*(*y* ≤ *q*) = *τ*. That is, we can consider the spread of the response in the form of a quantile;

$$\begin{array}{c} {\hat{Q}}_{\tau }\left(x\right)=\text{inf}\{y:\;\hat{F}\left(y|x=x_{i}\right)\geq \tau \}. \end{array}$$ (4)

When estimating the quantile function in Eq (4), it is observed that minimising an *l*_1_−loss function for a location estimator yields the median. An estimate of the *τ*-quantile is obtained by minimising a pinball loss function given in Theorem 1.

**Theorem 1.** *Let y* = {*y*_1_, *y*_2_, …, *y*_*m*_} ⊂ *R then the minimiser β*_*τ*_ *of* ∑*ρ*_*τ*_(*y*_*i*_ − *β*_*τ*_) *with respect to β for τ* ∈ (0, 1) *satisfies the following conditions*:

1. *The number of terms with y*_*i*_ < *β*_*τ*_ *is bounded from above by τm*.
2. *The number of terms with y*_*i*_ > *β*_*τ*_ *is bounded from above by* (1 − *τ*)*m*.
3. *If Pr*(*y*) *does not contain discrete components then* $\frac{m_{-}}{m_{+}}$ *converges to τ as m* → ∞.

*where*

$$\begin{array}{c} \rho_{\tau }=\rho \left(\tau_{q},y\right)=\{\begin{array}{cc} (1-\frac{q}{100})\left(\tau_{q}-y\right), & \text{for}\;\;y<\tau_{q}\\ \;\; & \;\;\\ \frac{q}{100}\left(y-\tau_{q}\right), & \;\;\;\;\text{for}\;\;y\geq \tau_{q}, \end{array}\} \end{array}$$ (5)

*is the pinball loss function*.

This means that solving the problem of minimising the expected quantile risk in RFs is equivalent to solving the following optimisation problem:

$$\begin{array}{c} \underset{\beta \in R}{\underset{︸}{\text{argmin}}}\sum_{i=1}^{n}w_{i}\left(x\right)\rho_{\tau }\left(y_{i}-\beta \right). \end{array}$$ (6)

To find the predictand at any quantile level, we redefine the quantile loss function by adopting the following definition by [21]:

$$\begin{array}{c} \rho \left(\tau \right)=\sum_{i=1}^{n}w_{i}\left(x\right)\{\sum_{i:u_{i}\geq 0}{\left(\tau u_{i}\right)}^{2}+\sum_{i:u_{i}<0}{\left(\left(\tau -1\right)u_{i}\right)}^{2}\}, \end{array}$$ (7)

where $u_{i}=y_{i}-{\hat{y}}_{\tau }\left(x\right)$.

### 2.2 The quantile generalised additive model

Another QR-based SI modelling framework is to look at the additive effects of its covariates. The framework developed by [49] takes smooth effects estimated by a GAM as inputs to a non-parametric QR. Ref. [50] explained that a GAM represents a method of fitting a smooth relationship between variables that can not easily fitted by standard linear or non-linear models. Ref. [51] added on saying that it is a generalized linear model with a linear predictor having a sum of smooth covariate functions. That is, it replaces the linear form ∑ *β*_*i*_*X*_*i*_ by a sum of smooth functions ∑ *s*_*i*_(*X*_*i*_). Thus, the focus in the additive regression model;

$$\begin{array}{c} E\left(Y|X\right)=s_{0}+\sum_{i=1}^{p}s_{i}\left(X_{i}\right) \end{array}$$ (8)

is to estimate the smooth functions. Since the QGAM takes these estimated smooth functions as its inputs then the GAM can benchmark the QGAM. A QGAM is a novel framework in climate science applications, and it does not need prior knowledge of the relationship structures of the response variable and its covariates. Ref. [52] introduced a learning rate $\frac{1}{\sigma }>0$ and positive semidefinite matrices **M** to a penalised pinball loss to estimate the regression coefficients on the QGAM by solving the following problem:

$$\begin{array}{c} {\hat{\beta }}_{\tau }\in \underset{\beta \in R^{d}}{\underset{︸}{\text{arg}\;\text{min}}}\sum_{i=1}^{n}\frac{1}{\sigma }\rho_{\tau }\{y_{i}-g_{i}\left(x_{i}^{T},\beta_{i}\right)\}+\frac{1}{2}\sum_{j=1}^{m}\eta_{j}\beta^{T}{\text{M}}_{j}\beta , \end{array}$$ (9)

where *η*_*j*_ are positive penalising smoothing parameters, $g_{i}\left(x\right)=\sum_{j=1}^{n}s_{j}\left(x\right)$ and the $s_{j}^{\prime }s$ are the additive smooth effects. The smooth effects are defined in terms of spline basis as

$$\begin{array}{c} s_{j}\left(x\right)=\sum_{k=1}^{K}\beta_{jk}B_{jk}\left(x_{j}\right), \end{array}$$ (10)

where *B* is the Bezier-spline basis function. Such a definition is deduced from the following theorem:

**Theorem 2.** *The function g* ∈ *W*^2^ *minimising*

$$\begin{array}{c} \sum \rho_{\tau }\{y_{i}-g\left(x_{i}\right)\}+\lambda V\left(g^{\prime }\right) \end{array}$$ (11)

*is a linear spline with knots at the points x*_*i*_, *i* = 1, 2, …, *d*, *where W is space of real functions.*

The learning rate determines the relative weight of the loss and penalty, while the matrices penalise the wiggliness of the corresponding smoothing effect. A scaled pinball loss replaces the pinball loss function called the extended log-f loss function (ELF);

$$\begin{array}{c} \rho_{\tau }^{*}\left(y-g\right)=\left(\tau -1\right)\frac{y-g}{\sigma }+\lambda \text{log}\left(1+e^{\frac{y-g}{\sigma }}\right),\;\;\;\;\lambda >0. \end{array}$$ (12)

The extended log-f loss enables efficient model fitting through smooth optimisation methods. The ordinary pinball loss function is piecewise linear and has discontinuous derivatives while the ELF loss leads to more accurate quantiles because it is an optimally smoothed version. Thus, it enables efficient model fitting through the use of smooth optimisation methods. Now, the regression coefficients being the solution to the problem in Eq (8) are obtained as a vector of maximum a posteriori (MAP) estimator, ${\hat{\beta }}^{T}$, for fixed *η* and *σ*_0_ by minimising the following penalised loss:

$$\begin{array}{c} V\left(\beta ,\eta ,\sigma_{0}\right)=\sum_{i=1}^{n}\text{log}\left[g\left(x_{i}\right),\sigma \left(x_{i}\right)\right]+\frac{1}{2}\sum_{j=1}^{m}\eta_{j}\beta^{T}M_{j}\beta . \end{array}$$ (13)

A stable estimation can be done by exploiting orthogonal methods for solving least squares problems. That is, the minimiser of Eq (11) corresponds to the minimiser of the following vector:

$$\begin{array}{c} V_{D}\left(\beta ,\eta ,\sigma_{0}\right)=\sum_{i=1}^{n}{\text{Dev}}_{i}\left\{\beta ,\sigma \left(x_{i}\right)\right\}+\sum_{j=1}^{m}\eta_{j}\beta^{T}{\text{M}}_{j}\beta , \end{array}$$ (14)

where ${\text{Dev}}_{i}\left\{\beta ,\sigma \left(x_{i}\right)\right\}=2\left[\text{log}\left\{g\left(x_{i}\right),\sigma \left(x_{i}\right)\right\}-\tilde{\text{II}}\right]$ is the *i*^*th*^ component of the deviance and $\tilde{\text{II}}$ is the staturated loss obtained by minimising the ELF w.r.t *g*. According to [52], iteratively minimising

$$\begin{array}{c} \sum_{i=1}^{n}w_{i}{\left(z_{i}-g_{i}\right)}^{2}+\sum_{j=1}^{m}\eta_{j}\beta^{T}M_{j}\beta , \end{array}$$ (15)

where

$$\begin{array}{c} z_{i}=g_{i}-\frac{1}{2w_{i}}\frac{\partial {\text{Dev}}_{i}\left\{\beta ,\sigma \left(x_{i}\right)\right\}}{\partial g_{i}}\;\;and\;\;w_{i}=\frac{\partial^{2}{\text{Dev}}_{i}\left\{\beta ,\sigma \left(x_{i}\right)\right\}}{\partial g_{i}^{2}} \end{array}$$

gives the estimates of the regression coefficients. [52] also suggested that the tuning parameters *η* and *σ*_0_ can be optimally selected through the Laplace Approximate Marginal Loss (LAML) criterion:

$$\begin{array}{c} L\left(\eta ,\sigma_{0}\right)=\frac{1}{2}V_{D}\left(\hat{\beta },\eta ,\sigma_{0}\right)+n\tilde{\text{II}}+\frac{1}{2}[\text{log}|X^{T}WX+S^{\eta }\left|-\text{log}\right|S^{\eta }{|}_{+}]-\frac{M_{p}}{2}\text{log}\left(2\pi \right), \end{array}$$ (16)

where $\hat{\beta }$ is the minimiser of the penalised loss, *W* is a diagonal matrix such that *W*_*ii*_ = *w*_*i*_, *M*_*p*_ is the dimension of the null space of *S*^*η*^ and |*S*^*η*^|_+_ is the product of its non-zero eigenvalues. Ref [53] developed the numeric stable formulas for computing the LAML and its derivatives.

### 2.3 Strengths and weaknesses of the QGAM and QRRF models

Table 1 comparatively presents the strengths and weaknesses of the QGAM and QRRF forecasting frameworks as non-parametric QR models. Non-parametric QR models share the following common advantages:

- They keep underlying assumptions as weak as possible to estimate conditional quantiles of the response in relation to many covariates [54].
- They provide more flexibility by modelling quantiles of the individual conditional response.
- The quantiles provide comprehensive information on the response distribution [55, 56], thus estimating a complete conditional distribution of the response variable.
- They are robust to outliers and give sparse solutions in high-dimensional data situations.

**Table 1:** Model comparisons.

Model	Strengths	Weaknesses
QGAM	1)Avoid inaccurate quantile estimates and inadequate coverage of the corresponding credible intervals by implementing the Bayesian methods of [57]. 2)Provides credible intervals to achieve adequate coverage for tail quantiles. 3) Bases QR on a smoothed version of the pinball loss. 4) The learning rate can depend on the vector of covariates [58]. 5) Renders model interpretable. and computationally tractable.	1) Additive in nature. 2) The large effect on the computational burden of the model from every interaction limits the number of covariates which may be added to the model [58]. 3) It involves tunning of parameters. 4) Layered nested optimisation can take a long time to converge.
QRRF	1) Able to represent forecasting uncertainty of the response as the probability distributions around a prediction interval. 2) Consistent and competitive interms of predictive power [19]. 3) Robust against overfitting. [7] 4) Every node is split into randomly chosen best subsets of covariates that performs very well. 5) Can learn highly irregular patterns in the data. with explicit regression	1) The probability density function of the future response variable cannot be obtained in a single QRRF model [59]. 2) Suffer performance problems in high-dimensional data [60]. 3) Response prediction is the mean of each leaf node response values of samples in that leaf which creates bias [59, 60]. 4) Does not give a clear model coefficients, thus limited interpretability [56].

### 2.4 Model validations

#### 2.4.1 Cross-validations

To obtain the best subset regression model, cross-validation of splitting 80% of the data set into training and 20% into test samples was done at first. That is, models were fitted using the training data frames, and prediction analyses were performed using the testing data frames.

#### 2.4.2 Root mean square error

The goodness of fit of the models was evaluated by measuring the deviations of the fitted from the actuals using computing the root mean square error (RMSE);

$$\begin{array}{c} \text{RMSE}=\sqrt{\frac{1}{n}{\left(y_{i}-\hat{y_{i}}\right)}^{2}}, \end{array}$$ (17)

where *y*_*i*_ is the actual observed SI and $\hat{y_{i}}$ is the corresponding model fitted value. The smaller the RMSE, the better the model fits the data.

### 2.5 Performance evaluations

#### 2.5.1 Mean absolute error

Mean absolute error (MAE) measures the average magnitude of errors in a set of predictions by providing a numerical value representing how far off the predictions are from the actual values. The MAEs in this study were computed as follows:

$$\begin{array}{c} \text{MAE}=\frac{1}{n}\sum \left|y_{i}-\hat{y_{i}}\right|, \end{array}$$ (18)

where the lower the score, the better the model.

#### 2.5.2 Mean absolute scaled error

The mean absolute scaled error (MASE) provides an interpretable measure of accuracy in predictive modelling. It is a good metric for comparing models trained on different datasets. It is one of the most appropriate metrics when the response has zero or near zero values. The metric is computed by dividing the trained model’s mean absolute error (MAE) by the MAE of the corresponding naïve mode as follows:

$$\begin{array}{c} \text{MASE}=\frac{\text{MAE}}{{\text{MAE}}_{naive}}. \end{array}$$ (19)

The naïve model predicts the value at a time point as the previous historical value. That is, MASE indicates the effectiveness of a forecasting model concerning a naïve model. As a result, a MASE greater than 1 means that the forecasting model is performing worse than the naïve benchmark; otherwise, it is better. As a result, in this study, we considered the forecasting model with a lower MASE to be a better model than the one compared to.

#### 2.5.3 Continuous ranked probability score

We also measured how the models predict the forecast distribution by averaging quantile scores over all values of *p* to give the CRPS. That is,

$$\begin{array}{c} \text{CRPS}\left(\hat{F_{p}},p\right)=\int_{-\infty }^{\infty }{\left(\hat{F_{p}}\left(y\right)-1_{p\leq y}\right)}^{2}dy, \end{array}$$ (20)

where $\hat{F_{p}}$ is the predictive cumulative density function and 1 is an indicator. A model with a lower CRPS predicted the whole forecast distribution better than one with a higher CRPS.

#### 2.5.4 Pinball loss function

In this study, we evaluated the sharpness of a QR-based model by computing its pinball loss score. It is considered as a special case of an asymmetric piecewise linear loss function defined as

$$\begin{array}{c} \text{Pinball}\left({\hat{Q}}_{y_{t}}\left(q\right),y_{t},q\right)=\{\begin{array}{cc} \left(1-q\right)({\hat{Q}}_{y_{t}}\left(q\right)-y_{t}), & \text{for}\;\;\;\;y_{t}<{\hat{Q}}_{y_{t}}\left(q\right)\\ \; & \;\\ q(y_{t}-{\hat{Q}}_{y_{t}}\left(q\right)), & \text{for}\;\;\;\;y_{t}\;\geq {\hat{Q}}_{y_{t}}\left(q\right), \end{array}\} \end{array}$$

where ${\hat{Q}}_{y_{t}}\left(q\right)$ is the predicted SI at the *q*^*th*^ quantile level and *y*_*t*_ is the actual SI. A pinball loss score closer to zero is much desired.

#### 2.5.5 Winkler score

The Winkler score is a trade-off between coverage and the prediction interval width. It is the length of the prediction interval plus a penalty if the observation is outside the interval defined as

$$\begin{array}{c} W_{\alpha ,t}=\{\begin{array}{cc} \left(u_{\alpha ,t}-l_{\alpha ,t}\right)+\frac{2}{\alpha }\left(l_{\alpha ,t}-y_{t}\right), & \text{for}\;\;\;y_{t}<l_{\alpha ,t}\\ (u_{\alpha ,t}-l_{\alpha ,t})\;\;\;\;\;\;\;\;\;\;\;\;\;\;\;\;\;\;, & \text{for}\;\;\;\;l_{\alpha ,t}\;\leq y_{t}\leq u_{\alpha ,t}\\ \left(u_{\alpha ,t}-l_{\alpha ,t}\right)+\frac{2}{\alpha }\left(y_{t}-l_{\alpha ,t}\right), & \text{for}\;\;\;y_{t}<u_{\alpha ,t}, \end{array}\} \end{array}$$

where [*l*_*α*,*t*_, *u*_*α*,*t*_] is the (100 − *α*)% prediction interval at time t. We compared the models by finding the one with a smaller Winkler score. The smaller the Winkler score, the narrower the prediction interval.

#### 2.5.6 Coverage probability

Coverage probability (CP) was computed by running numerous samples, and a wide range of possible outcomes was generated for each sample. Then, this range of possible outcomes was compared to the actual value to see if they properly accounted for it in its range. That is, if the probability of a randomly chosen observed response value had a probability of at least 0.95 to fall within the forecasted 95% prediction interval, then the model was reliable, well-calibrated or unbiased. We considered a model with a higher CP score to be a more reliable one.

## 3 Simulation study

### 3.1 The data-generating process

To evaluate the finite sample properties of the QRRF model and QGAM and test for equal accuracy of their forecasts, we used simulations of multivariate data-generating processes. The QGAM was found to be the near-best additive model to predict SI by [6]. Features considered in the current study are the ones identified by [46]. The current study also used the R programming software; version 4.3.2 (2023-10-31 ucrt) as the computational tool. The first activity was to fit probability distributions to the variables understudy. The covariance matrix of the variables was computed using their fitted probability distributions. The covariance matrix was computed with the help of the “JWileymisc” R programming package. We combined the computed covariance matrix and the extracted distributional shape parameters from the fitted probability distributions to generate 50 data frames of 5000 data points. The code can be found at https://github.com/amonmasache/Non-parametric-QR-Models and the simulated data frames at https://github.com/amonmasache/phd-simulated-data.

### 3.2 Forecasting accuracy evaluations

The models were then trained on 80% of each generated data set. Evaluation of the pinball loss score, CRPS and the MASE score was done on independent test data frames, which were 20% of each generated data set. The split of the data set was done to cross-validate the models and [6] found 20% forecasting horizon to be an ideal data split when forecasting SI. We focused the simulation study on evaluating the pinball loss score as the most important error measurement for QR models. We also included the MASE metric in the comparison investigation for its strengths on variables with zero values in their observations. The QGAM had significantly lower minimum, mean and maximum scores on the two forecasting accuracy metrics than the QRRF model (Table 2).

**Table 2:** Summary statistics of computed metrics.

	QGAM			QRRF		
Statistic	PinBall	CRPS	MASE	PinBall	CRPS	MASE
Min	**0.161069**	0.781578	0.192208	0.324651	**0.781125**	**1.158690**
Max	**0.176650**	0.849332	**0.219781**	0.337189	**0.848577**	1.294265
Mean	**0.168011**	0.809772	**0.207209**	0.330317	**0.809382**	1.222083
Var	1.23E-05	0.000295	**4.58E-05**	**1.01E-05**	**0.000292**	0.000942

*Entry in bold is the better metric value between the QGAM and QRRF for a particular statistic.

**Fig 3:**
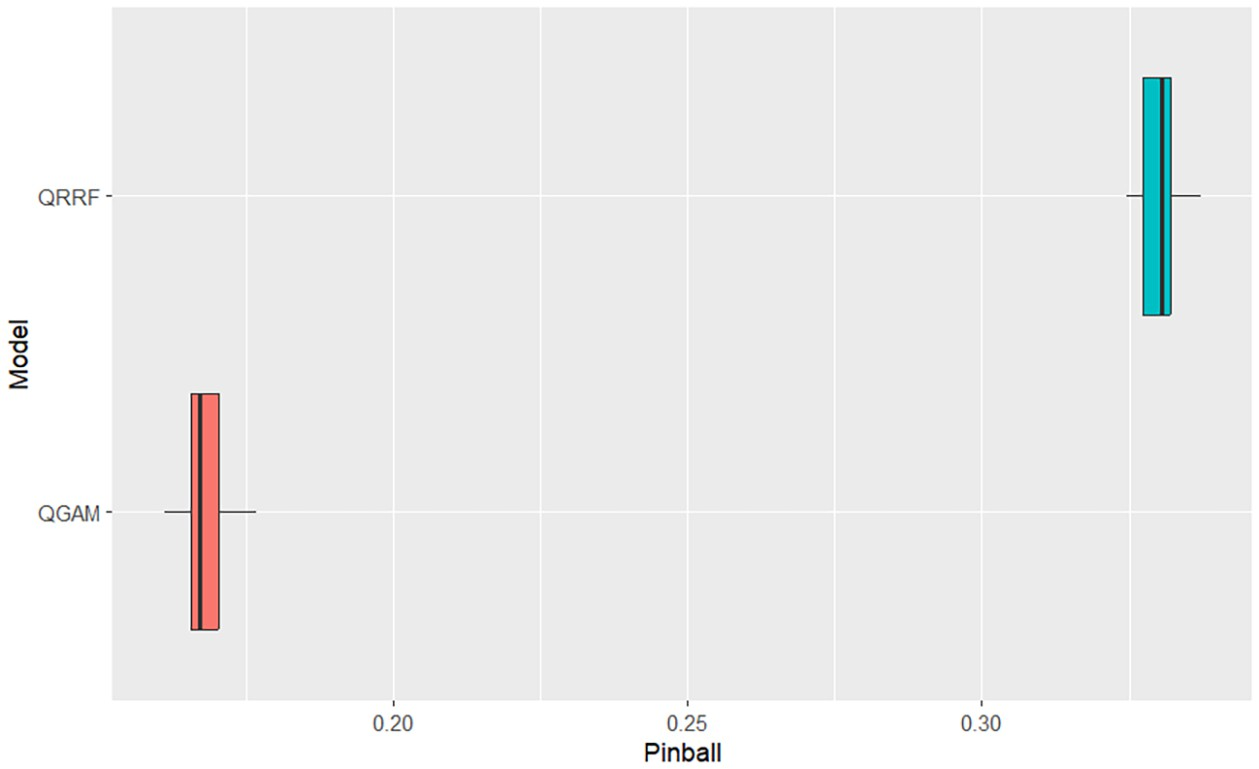
QRRF vs QGAM Pinball scores box plot comparison.

**Fig 4:**
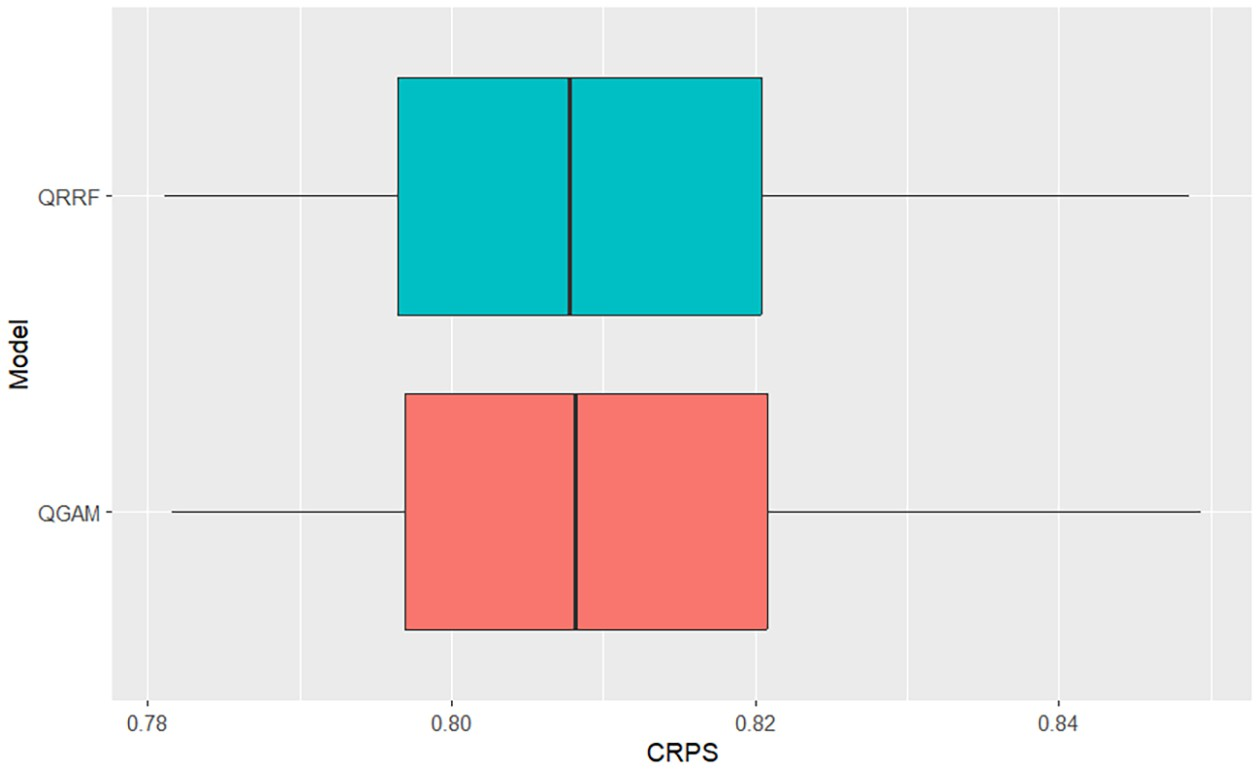
QRRF vs QGAM CRPS box plot comparison.

**Fig 5:**
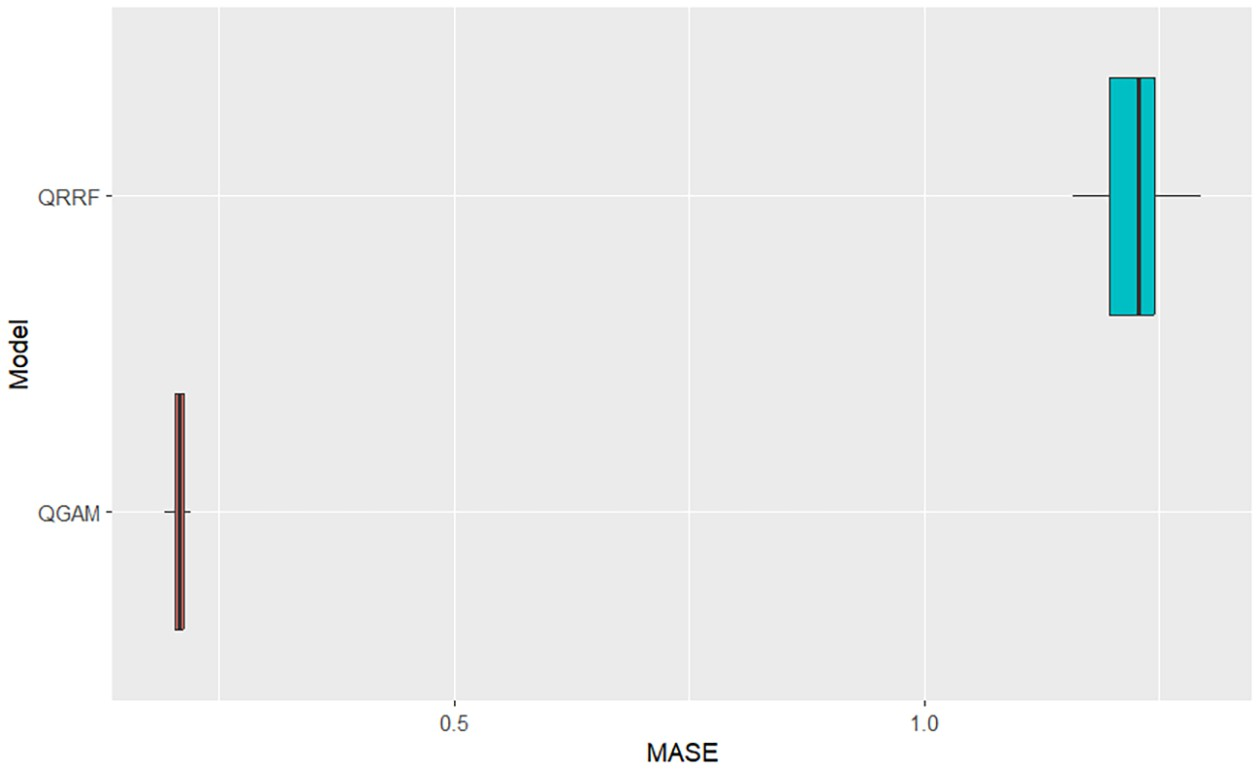
QRRF vs QGAM MASE scores box plot comparison.

The simulation results indicate that the QGAM had better forecasting performance than the QRRF model. The QGAM framework avoids inaccurate quantile estimates and inadequate coverage of the corresponding credible prediction intervals by implementing the Bayesian methods of [61]. We also argue that the smoothed version of the pinball loss, the ELF loss function, increases the conditional quantile prediction accuracy of the QGAM over other non-parametric QR models. Ref. [58] highlighted that the learning rate introduced by [52] can depend on the vector of covariates giving the QGAM a lower prediction error than the QRRF as indicated by the MASE scores. However, the CRPS summary statistics indicated that the two models predicted the same solar irradiance distribution. They had similar accuracy in predicting the forecast distribution. We note that both the QGAM and QRRF apply the same concept, the method of quantiles, to predict the forecast distribution through conditional quantiles of SI in relation to its covariates. This makes both of the non-parametric QR-based models under comparative investigation flexible, robust to outliers and give sparse solutions in the SI data being forecasted in this current study. Graphical analysis of box plots in Figs 3–5 verifies results in Table 2. They had similar behaviour on distributional forecasts, as shown in Fig 4.

The QGAM had less variability in the MASE scores because its box plot was much narrower than the QRRF. However, the pinball loss and MASE scores box plots demonstrate significantly better performance of the QGAM than the QRRF. Though the pinball loss box plots show approximately the same variability, the box plot for the QGAM is on the lower values of the graph. Pinball loss scores should be as low as possible. The MASE box plot of the QGAM is also on the lower values like the pinball loss box plot. The results agree with the review of the literature, which shows that the QRRF model suffers performance problems in high-dimensional data situations like the SI data under study. We also note that [59] argued that a single QRRF model run cannot give the probability distribution function of the future response variable thereby explaining the performance exhibited by the model. In addition, decision tree-based models may change the prediction accuracy each time a training is repeated.

## 4 Illustration with Windhoek SI data

### 4.1 Data exploration

Raw data from the Namibia University of Science and Technology radiometric station was accessed from the Southern Africa Universities Radiometric Association Network website (www.sauran.ac.za). The SI data was collected as an hourly averaged GHI data set from March 2017 to June 2019. Missing observations were imputed using the “Hmisc” package developed by [62] and the raw data was cleaned to the data set found at https://github.com/amon.masache/Non-parametric-QR-Models. Skewness of 0.132 and kurtosis of -1.196 in Table 3 show that GHI is abnormal.

**Table 3:** Summary statistics for GHI.

Min	1stQu.	Median	Mean	3rdQu.	Max.	Skewness	Kurtosis
0.1000	157.800	501.800	490.00	772.100	1251.300	0.132	-1.196

**Fig 6:**
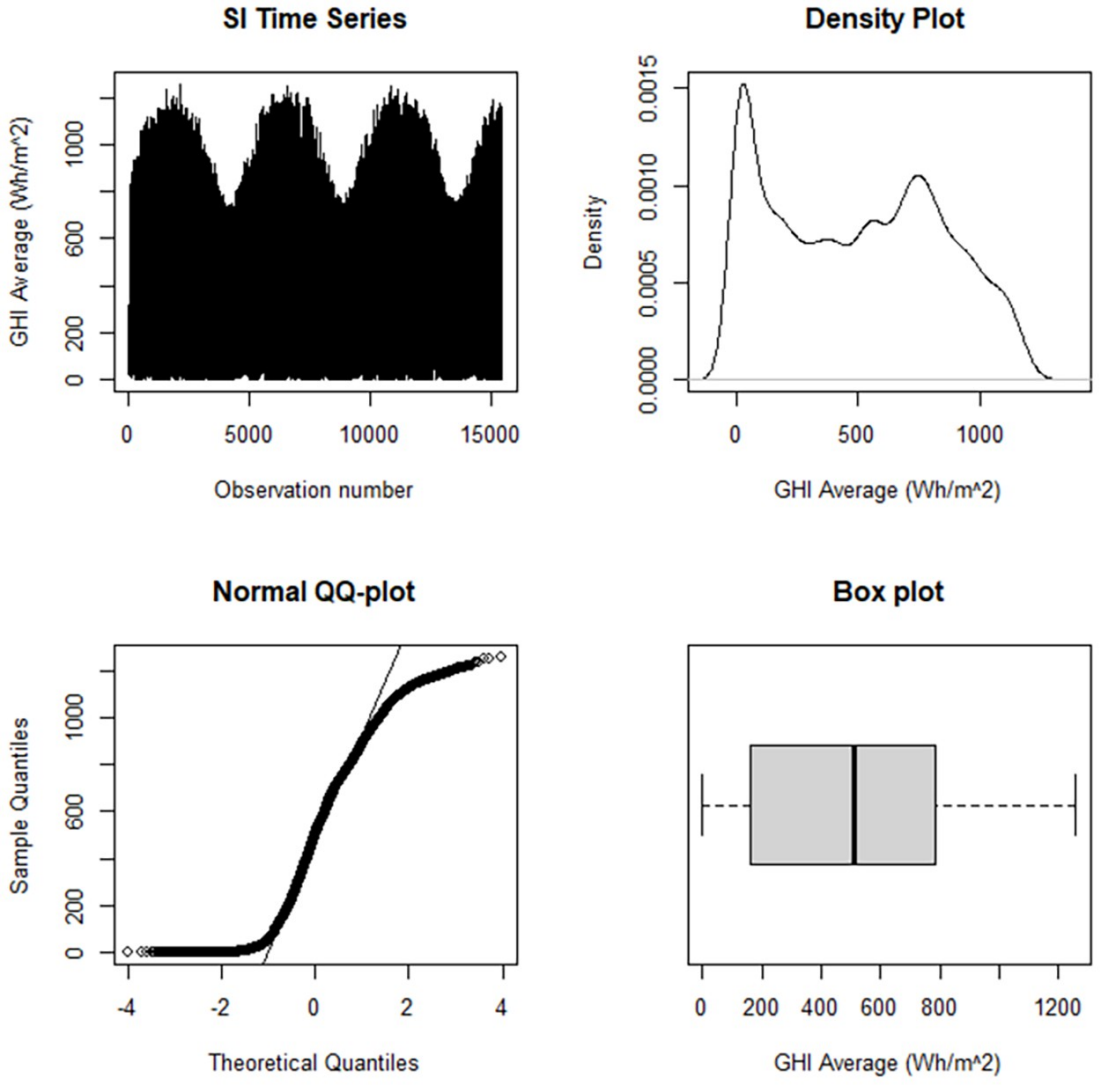
Diagnostic plots for GHI.

The data exhibited an asymmetric heavily tailed distribution, as shown by the density plot and box plot in Fig 6. The density plot also shows an unknown probability distribution function. The Jarque-Bera test had a p-value less than 0.05, confirming that solar irradiation is not normally distributed.

### 4.2 Variable selection

To model the trend in the time series, lag1 and lag2 of GHI were included in the data frame. Lasso via hierarchical pairwise interactions selections, using the ‘hermit’ package by [63] was applied to the covariables and results in Table 4 were obtained. The significant main effects were hour, temperature (Temp), wind speed (WS) and barometric pressure (BP). Non-zero entries in Table 4 indicate that the variable (main effect column) or the interaction (the other columns) effect is significant.

**Table 4:** Lasso heirachichal pairwise interactions selections.

Variable	Main Effect	Hour	Temp	RH	WS	BP	Lag1	Lag2
Hour	-164.96	-83.90	-1.028	4.73	0.00	0.00	-88.13	0.00
Temp	19.25	-1.03	0.00	0.00	-0.54	0.00	-4.77	0.00
RH	-20.17	4.73	0.00	-3.26	0.00	0.00	-8.08	-8.46
WS	7.06	0.00	-0.54	0.00	0.00	0.00	0.00	0.00
BP	-0.31	0.00	0.00	0.00	0.00	0.31	0.00	0.00
Lag1	250.30	-88.13	-4.77	-8.08	0.00	0.00	0.00	0.74
Lag2	-13.93	0.00	0.00	-8.46	0.00	0.00	0.74	13.93

A Kwiatkowski-Phillips-Schmidt-Shin (KPSS) test was done to check on the stationarity of the covariates. The KPSS test is the most appropriate for large samples and among the most effective stationarity tests available. The KPSS test results in Table 5 indicate that all covariates were stationary because the KPSS p-values were all less than 0.05.

**Table 5:** KPSS test p-values.

Variable	GHI	Hour	Temp	RH	WS	BP	Lag1	Lag2
p-value	0.034	0.021	0.010	0.010	0.038	0.010	0.035	0.036

### 4.3 Model fitting and evaluations

The RFs modelling framework was considered the benchmark model and was fitted using the “randomForest” package in R programming software developed by [64]. On the other hand, the “quantregForest” package was used to analyse the QRRF model. There were 500 trees grown and 5 variables were tried on each split on the two decision tree-based models. The QGAM was fitted using the “mgcViz” package developed by [65] through the following expansion:

$$\begin{array}{ccc} {\text{GHI}}_{t} & = & s(\text{Hour},bs=“cc”)+s(\text{Lag1})+s(\text{Lag2})+s(\text{Temp})+s(\text{RH})+s(\text{WS})+s(\text{BP})\\ \; & \; & +s(\text{Hour}\times \text{Temp})+s(\text{Hour}\times \text{RH})+s(\text{Hour}\times \text{Lag1})+s(\text{Temp}\times \text{WS})\\ \; & \; & +s\left(\text{Temp}\times \text{Lag1}\right)s\left(\text{RH}\times \text{Lag1}\right)+s\left(\text{RH}\times \text{Lag2}\right)+s\left(\text{Lag1}\times \text{Lag2}\right)+\epsilon_{t}, \end{array}$$ (21)

where *s*(.) is a smooth function defined as in Eq (9) and *bs* = “cc” is the cyclical effect of time measured in hours. When running Eq (20), the software gives the estimated effective degrees of freedom (edf) for the approximated smooth functions (see Table 6) because it is a non-parametric model. It took a notable long time to run the QGAM code to give the results in Table 6. All smooth functions had significant effects on GHI except the smooth function of the interaction effect between temperature and wind speed. All models were valid to fit the data because they all had Theil’s U statistics less than 1 (Table 7). That is, the non-parametric models fitted the data better than their corresponding naïve models. Cross-validation correlation results show that the QRRF model slightly overfitted the data because the training data set has a higher correlation coefficient (r = 0.996) than that of the testing data set (r = 0.965). In contrast, the RFs and QGAM models neither overfitted nor underfitted the data because the correlation coefficients of the training and testing data sets were approximately equal. However, the GAM underfitted the data, its testing correlation is less than that of the training.

**Table 6:** QGAM smooth function estimates.

Smooth Function	edf	Chi-square	p-value
*s*(*Hour*)	7.801	1563.795	< 2.0 × 10^−16^
*s*(*Lag*1)	8.753	4440.583	< 2.0 × 10^−16^
*s*(*Lag*2)	8.250	304.901	< 2.0 × 10^−16^
*s*(*Temp*)	7.454	96.319	< 2.0 × 10^−16^
*s*(*RH*)	6.235	106.792	< 2.0 × 10^−16^
*s*(*WS*)	3.539	14.341	1.2 × 10^−2^
*s*(*BP*)	4.086	97.151	< 2.0 × 10^−16^
*s*(*Hour* × *Temp*)	5.633	28.963	1.4 × 10^−4^
*s*(*Hour* × *RH*)	6.536	47.530	< 2 × 10^−16^
*s*(*Hour* × *Lag*1)	7.735	2076.930	< 2 × 10^−16^
*s*(*Temp* × *WS*)	2.496	6.144	1.2 × 10^−1^
*s*(*Temp* × *Lag*1)	3.579	71.317	< 2.0 × 10^−16^
*s*(*RH* × *Lag*1)	8.344	682.031	< 2.0 × 10^−16^
*s*(*RH* × *Lag*2)	7.423	169.665	< 2.0 × 10^−16^
*s*(*Lag*1 × *Lag*2)	5.317	20.580	4.6 × 10^−3^

**Table 7:** Model validations.

(a) Model fit evaluation.		(b) Cross-validation correlations.		
Model	Theil’s U	Model	Training	Testing
RF	0.3217	RF	0.966	0.964
QRRF	0.3602	QRRF	0.996	0.965
QGAM	0.1878	QGAM	0.966	0.967
GAM	0.1760	GAM	0.968	0.938

**Fig 7:**
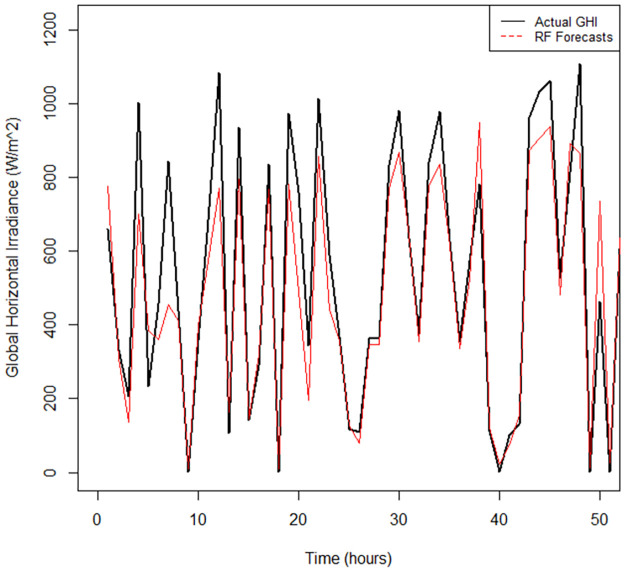
RFs model fitted (in red) vs actual (in black) time series plot.

**Fig 8:**
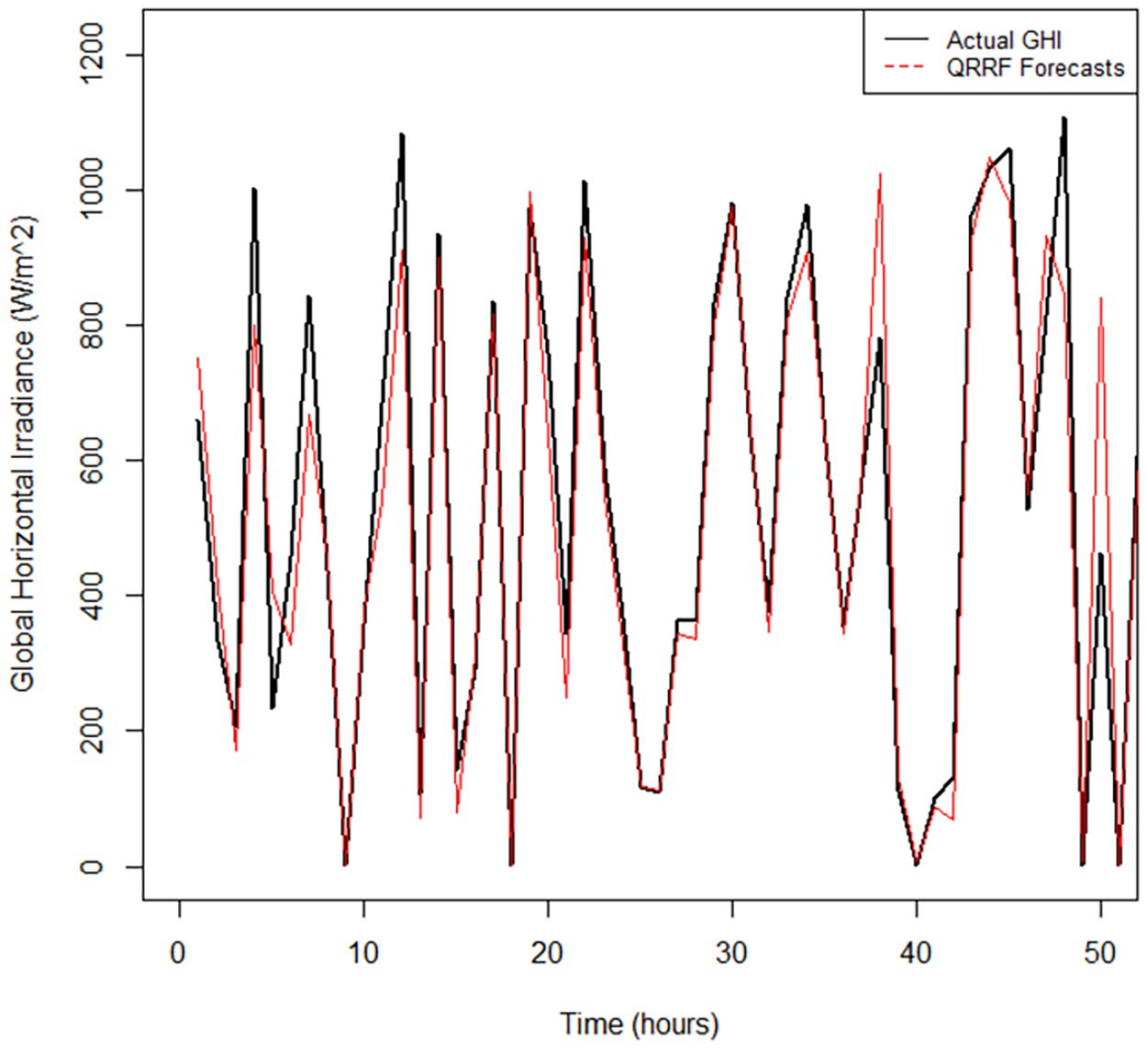
Fitted vs actual time series plots for the QRRF model.

**Fig 9:**
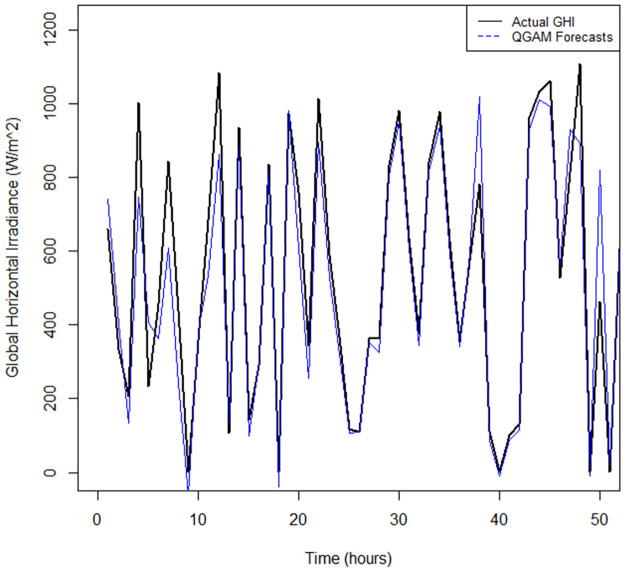
Fitted vs actual time series plots for the QGAM model.

The time series plot in Fig 7 shows that the RFs model did not fit the data closely to the actual values compared to the QRRF and QGAM fitted time series (see Figs 8 and 9). There are notable gaps between the forecast time series and that of the actual observed GHI values. The time series fitted by the QGAM and QRRF models compared very closely with the observed data. Apart from indicating how excellently the QGAM and QRRF fit the data, the results demonstrate the great forecasting accuracy the two non-parametric QR models have. The RMSE metric was also used to find the model that best fitted the data. The QGAM had a near lowest RMSE, followed by QRRF, and the RFs model had the largest RMSE (Table 8). This means that the QGAM fitted the data nearly the best while the RFs model fitted the data the worst among the three models compared. The MAE in Table 8 also showed that the QRRF had slightly the closest predictions to the observed GHI (MAE = 11.11), while the RFs model had the furthest predictions from the actual values (MAE = 14.47). Since all models had MASE scores less than 1, all of the models forecasted GHI better than their corresponding naive models when trained in the data. However, the RFs model had the highest MASE(=0.1399) score, while QRRF had the lowest (=0.1177) score. The QGAM had a MASE score of 0.1232, close to the MASE score of the QRRF. It can be deduced that the QRRF outperformed its corresponding naïve model better than how the QGAM outperformed its corresponding naïve model. Both the QRRF and QGAM frameworks were better than the GAM and RFs models when considering the forecasted distributions of GHI because the GAM and RFs models had the largest CRPS. The GAM model predicted the forecast distribution worse than any of the compared models, and the QGAM predicted the forecast distribution to be nearly the best among the three models. Results show that the GAM model had the worst forecasting performance metric values among the non-parametric models fitted in this study.

A skill score of 0.001 which is greater than 1 (Table 8) confirms that and shows that hybridising the RFs model with QR improved the accuracy of the RFs modelling framework. Ref. [51] also demonstrated that the QGAM framework is an improvement of the GAM. The GAM and RFs models go only as far as forecasting the conditional mean distribution. Such forecasts are affected by outliers which are often present in SI data. Conditional mean forecasting can over- or under-estimate extreme values in the data samples. Since the GAM and RFs models are limited to the conditional mean, then the two models were excluded from further analyses but were done on the QRRF and QGAM models.

**Table 8:** Out-of-sample diagnostic evaluations.

(a) Metric evaluations.					(b) QRRF vs RF.	
Model	RMSE	MAE	MASE	CRPS	skillscore	skillscore.sd
RF	24.866	14.47	0.1399	206.49	0.0011103634	0.0006542398
QRRF	21.634	**11.11**	**0.1177**	205.46		
QGAM	**21.233**	11.78	0.1232	**205.39**		
GAM	87.638	50.04	0.1350	205.79		

*Entry in bold is the best metric value.

**Fig 10:**
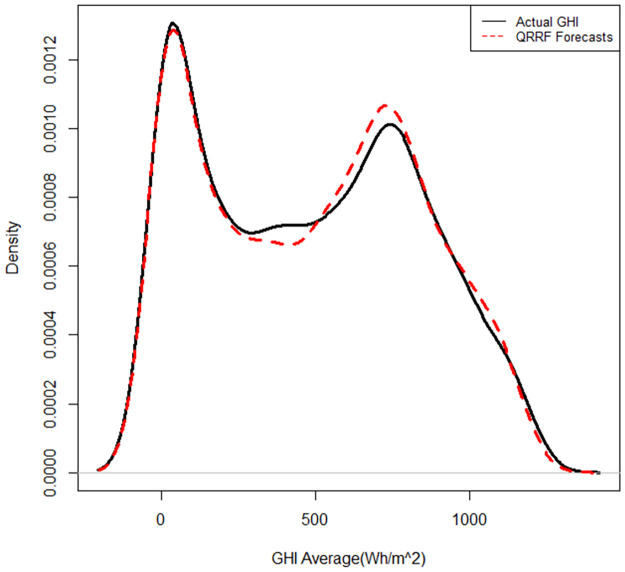
Fitted vs actual density plot for the QRRF model.

**Fig 11:**
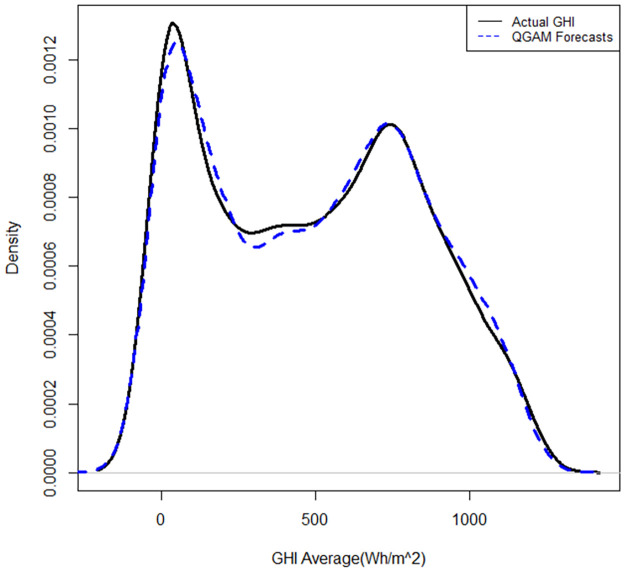
Fitted vs actual density plot for the QGAM model.

The QRRF and QGAM models fitted the densities very closely to the actual densities, as shown in Figs 10 and 11.

**Fig 12:**
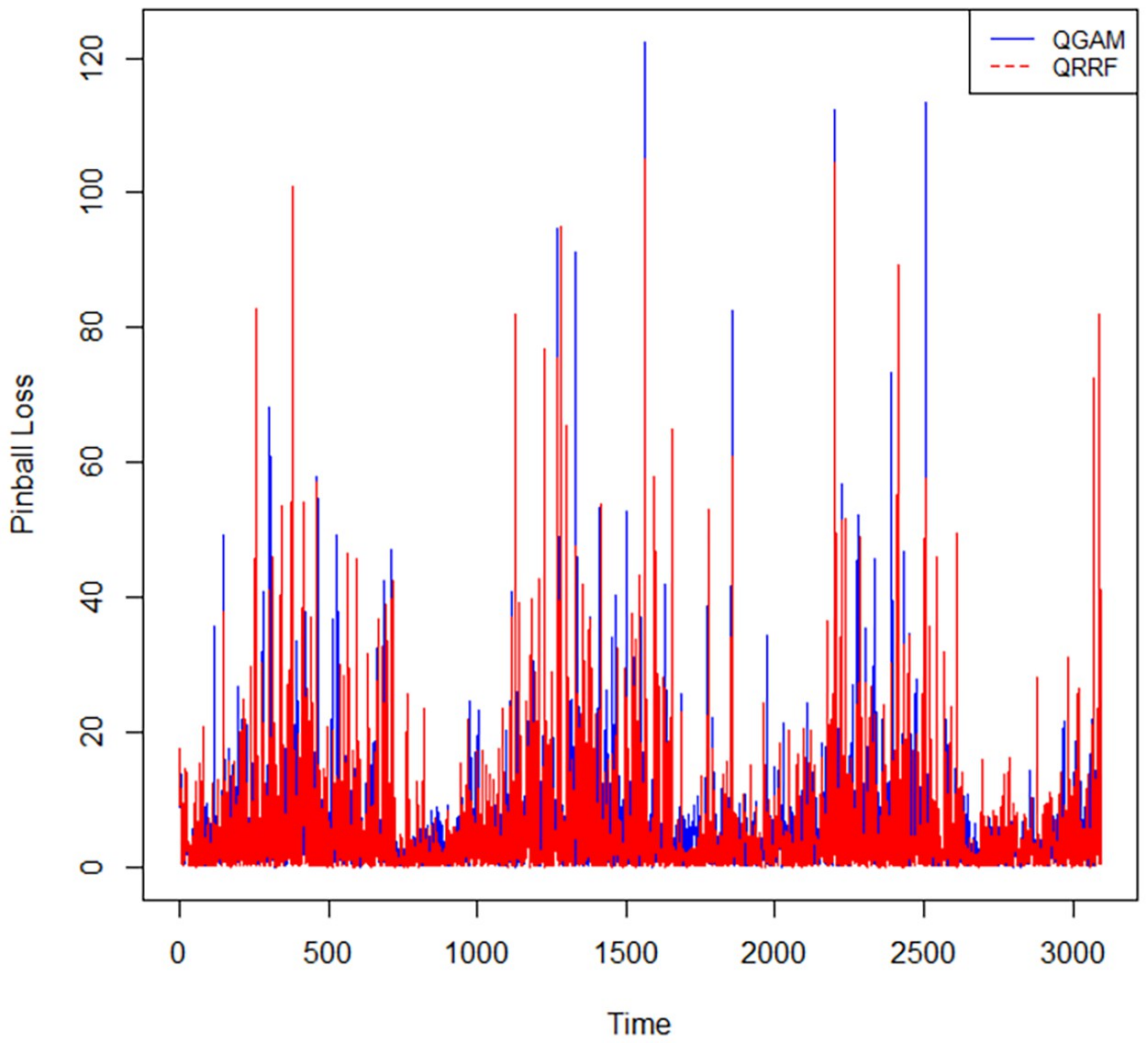
Pinball losses over time.

However, the density fitted by the QGAM looks better than that of the QRRF, especially on the turning points of the density curves and around the zero value of SI. The predicted curves of the QRRF are further away from the actual than those predicted by the QGAM, especially on the turning points. Results in Table 9 show that QRRF had a slightly better pinball loss score than the QGAM. However, the graph in Fig 12 suggests that the QGAM and QRRF models had almost the same pinball loss scores because the two models have almost the same number of longer spikes than the other.

**Table 9:** QRRF and QGAM performance comparisons.

Model	Pinball loss	Winkler Score	CP
QGAM	5.889	**0.828**	0.9535
QRRF	**5.555**	0.831	**0.9493**

*Entry in bold is the best metric value.

The QGAM had a slightly lower CP score than the QRRF (Table 9), indicating that the QGAM had slightly better reliability and unbiasedness. The QGAM also had a slightly better trade-off between coverage and the prediction interval width because its standardised Winkler score was slightly lower than that of the QRRF model.

**Fig 13:**
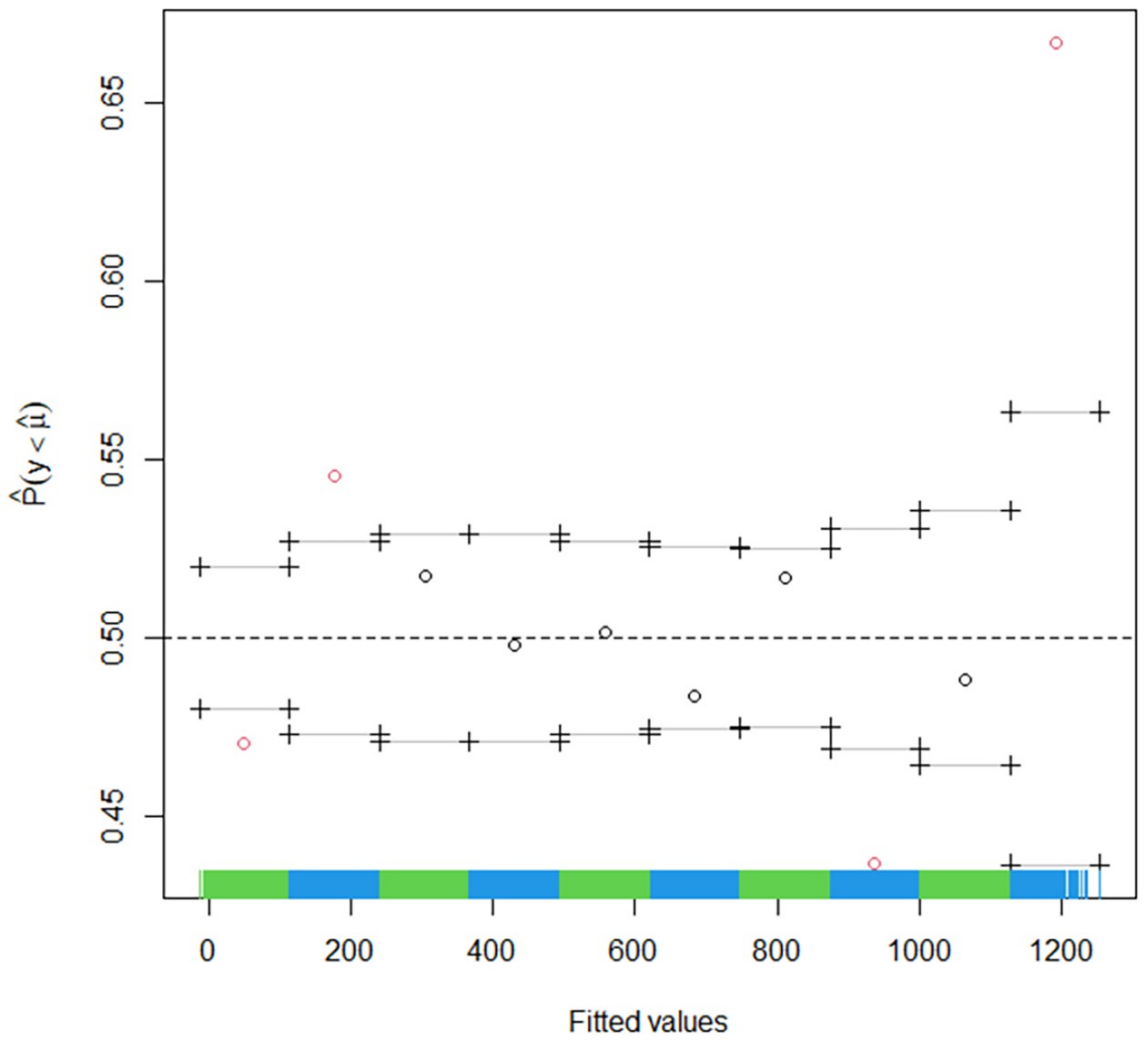
QGAM estimated prediction interval.

**Fig 14:**
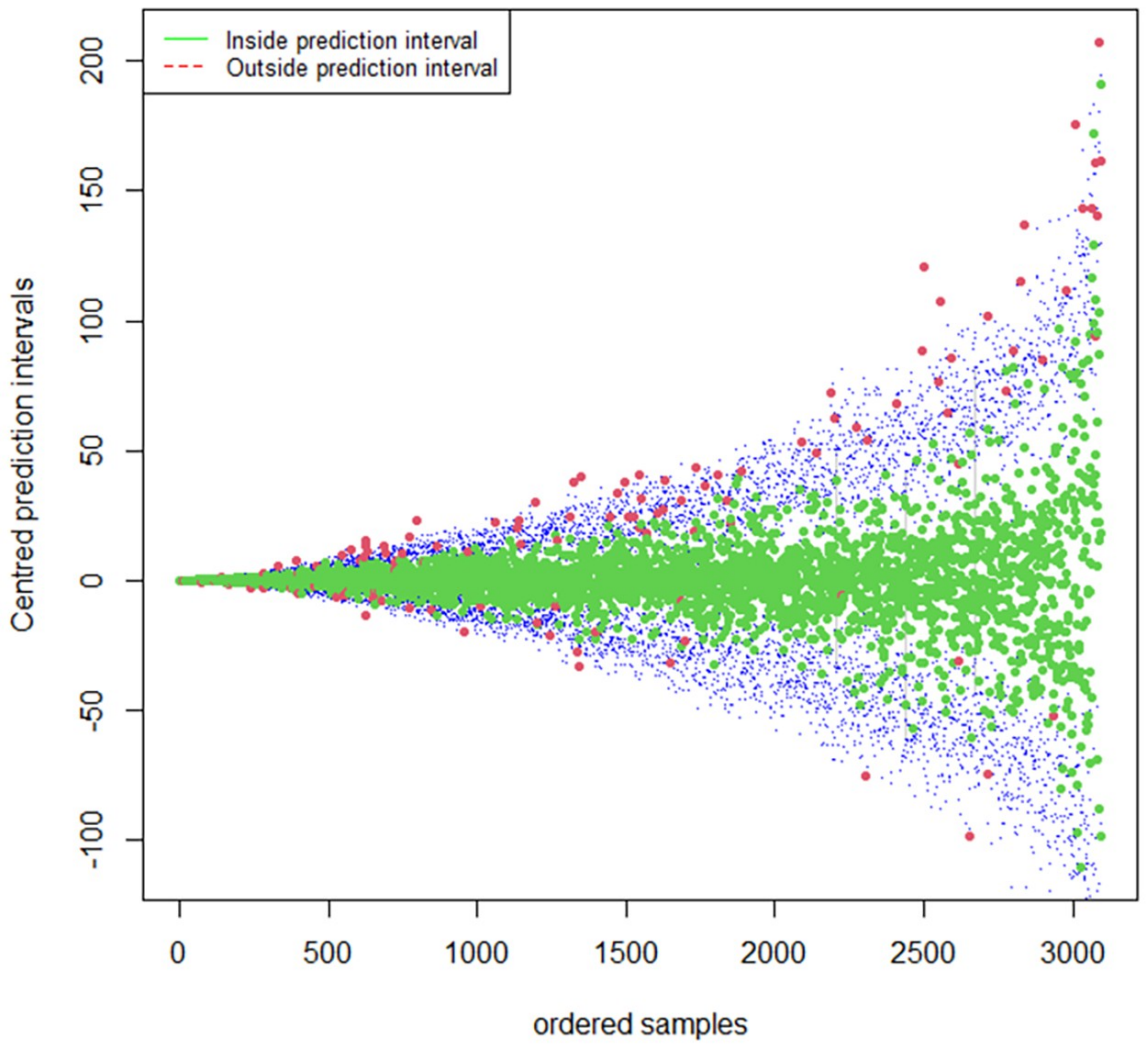
QRRF estimated prediction interval.

The 95% prediction interval from the QGAM is in Fig 13, and the red small circles indicate that the fitted quantile might be deviating too much from the actual quantile. On the other hand, Fig 14 shows the 95% prediction interval from the QRRF model.

**Fig 15:**
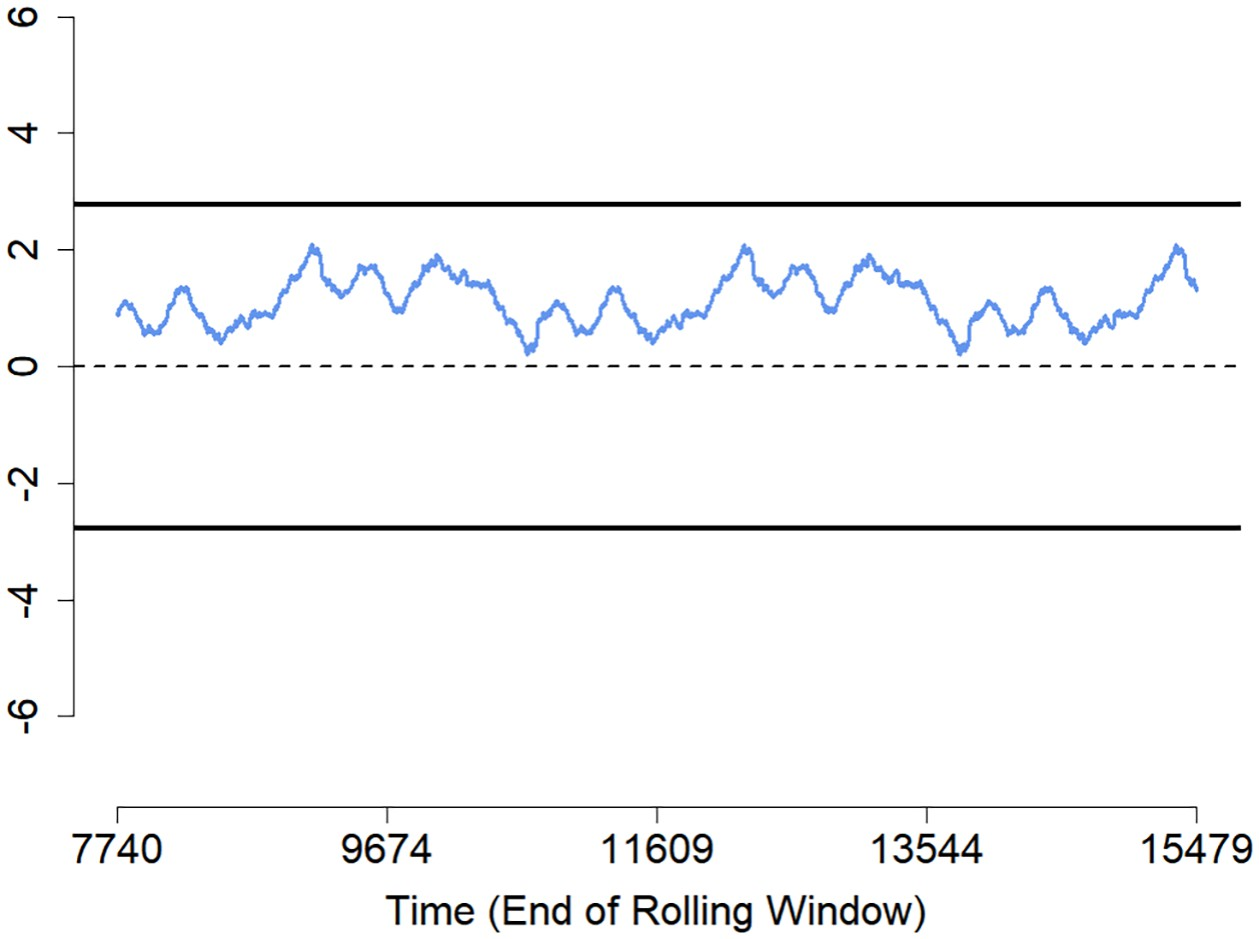
Fluctuation test plot.

The fitted points in green are inside, and those in red are fitted points outside the prediction interval. The prediction interval is indicated by blue points on the bottom for the lower limits and on the top for the upper limits. If we compare the two prediction intervals within the first 1200 actual observations, then the QRRF prediction interval seems to have slightly more points outside the prediction interval than the QGAM prediction interval plot. As a result, the QGAM had a slightly better prediction interval than the QRRF model. We also tested for the statistical significance of the two models using the Diebold-Mariano test to check whether the two models have the same predictive accuracies. A p-value of 0.2943 on a null hypothesis, ‘The two models have the same forecasting accuracies.’ against the alternative hypothesis, ‘The two models have different forecasting accuracies’, was obtained. This means that the null hypothesis could not be rejected, and we deduced that the two models had the same accuracy when predicting solar irradiation. The Diebold-Mariano test was done on the covariate stationarity assumption, validated in subsection 4.2. The Diebold-Mariano test results confirmed the slight differences in the metric values from the QGAM and QRRF models. A fluctuation test was done to analyse whether the ranking of two forecasts was stable over time using a null hypothesis that both QRRF and QGAM forecasting models performed equally well at all time points. The results are shown in Fig 15. Since the fluctuations are within the confidence bands, their performances did not differ by at least one-time point. It also means there was stability in the relative performance of the two models over time.

## 5 Discussions

A simulation study was conducted to compare the accuracy of the QGAM and QRRF models. They had the same accuracy only on the forecasted probability distribution. Even though [21] highlighted that the QRRF model prediction uses the entire CDF information and is a non-equal-weight approach, the model did not have a better CRPS than the QGAM in this study. Both the QGAM and QRRF models are non-parametric QR-based models on which quantiles provide comprehensive information on response variable distribution as a function of the covariates. In addition, they provide more flexibility by modelling quantiles of the individual conditional response. So, they use the same concept of minimising the pinball loss function when estimating conditional quantiles thereby predicting similarly the conditional distribution. More simulation results suggested the superiority of the QGAM on both the pinball and MASE scores. The QGAM uses the minimised smoothed version of the pinball loss function, the ELF, that applies a series of smooth optimisation methods as efficient and accurate parameter tuning processes. The optimisation methods include the LAML criterion which can be computed by numeric formulas of [53] to give stable estimates. Thus more accurate quantile estimation. The ELF minimisation also includes tuning the learning rate which refines the optimisation process. The series of smooth optimisations includes minimising the regularised empirical risk which can account for the better MASE score. The regularised empirical risk is defined through a very efficient reproducing Kernel Hilbert space (RKHS) norm and connected with covariate spaces to formulate a minimisation problem that can be easily solved with a standard quadratic programming code [55]. The maximum a posteriori estimation of the regression coefficients through smooth optimisation methods can also be used to explain better simulation results from the QGAM than the QRRF model. In addition, the estimation process developed by [52] exploits orthogonal methods to give stable estimated regression coefficients. The inferiority of the QRRF from the simulation results can be accounted for by [10], who explained that the QRRF model’s prediction intervals are often wide. This is because each conditional response distribution is separately estimated using a relatively small amount of data local to a point in the predictor space at which the prediction interval is needed. The QRRF model estimates separate conditional response distributions for all new cases. In addition, tree-based models cannot predict values not in the training data set. They are limited to the range of values of the training data set. Ref. [10] also discovered that the prediction interval from a QRRF model sometimes over- or under-cover the target response values. Worse, the model tends to have Type I and Type II coverage rates that deviate from the nominal level.

Similar results were obtained when the two non-parametric QR-based models were applied to real-life data. The illustration study demonstrated that the two tree-based models and the additive effects model were excellent fits of non-parametric models to the solar irradiation data. That is, our results agree with researchers like [10, 26, 27] that non-parametric models are very good at learning data with unknown distributions and relationship structures between the response and covariates. Apart from the QRRF model slightly overfitting the data, both the QGAM and RFs models fit well. We attribute the overfit to the highly variable estimators of the conditional solar irradiation distribution in QRRF. However, the QRRF and QGAM were better than the RFs model on all out-of-sample metric evaluations because RFs produce point forecasts that are not accompanied by information about how far the predictions may be from the true response values. This argument also accounts for the RFs improvement through hybridisation with QR modelling and these results agree with findings from [32]. The proposed hybrid, the QRRF model, became a means of obtaining a tree-based prediction interval through estimating the conditional distribution. The QRRF model delivered an approximation of the full conditional distribution [24]. So, it is a generalisation of RFs because both are a set of classification and regression trees. We also note that predictive quantiles are directly obtained from the CDF’s average CDF estimated from the ensembled trees.

The RMSE metric measures the deviations from the actuals. That is, assessing how effectively a model is learning the data. If the model can learn the data well, it can predict the test data with small deviations. Ref. [52] improved the learning effectiveness of a QGAM by introducing a learning rate and positive semidefinite matrices to a penalised pinball loss. The components are penalties on the model and control the deviations as the model predicts the test data. Thus, the mean square error is minimised by applying the marginal loss minimisation when selecting the loss smoothing parameters. Thereby, the lowest RMSE in this study. On the other hand, we account for the near lowest MAE in terms of how the QRRF combines the estimated OOB prediction errors empirical distribution with the RFs response distribution. In addition, the QRRF is linked to the analogues method in the sense that it is another way to find the closest observations given a set of predictors [26].

## 6 Conclusions

The current study focused on comparatively investigating the forecasting performance of the QRRF and QGAM as new predictive models in SI studies. The study provided a proof-of-concept for the use of the QRRF model to forecast SI data. At first, covariates were selected using the lasso via the hierarchical pairwise interactions method after confirming that SI is heavily tailed, contains outliers, and is abnormal. Simulation results showed that the non-parametric QR frameworks introduced could predict the same distribution of solar irradiation. However, from the evaluation of pinball loss and MASE scores and other metric evaluations, we can conclude that the QGAM is a better non-parametric QR model than the proposed QRRF model for predicting solar irradiance. Similar results were obtained when the models were applied to real-life data. That is, we recommend the application of the QGAM and QRRF models when predicting the distribution of solar irradiance from Southern Africa but with a preference for the QGAM. We also conclude that hybridising an RFs model with QR improves the forecasting accuracy of the decision tree-based model when predicting SI.

The modelling framework proposed in this study is important to the renewable energy industry, which requires accurate forecasts of the intermittent solar power. The framework can be extended to other renewable energy sources because they have similar meteorological characteristics. Such modelling frameworks improve the stability of electricity generation and management in an effective, environmentally secure way. Renewable sources of energy studies favour the future economic prosperity of the Southern African region. The tree-based QR model proposed in this study is robust and, as such, can easily be adapted to forecasting different key response variables. However, decision tree-based models change slightly their prediction accuracies each time training is repeated. In addition, they do not give a clear model with explicit regression coefficients. As a result, decision tree-based models have limited interpretability. On the other hand, the layered nested optimisation on the QGAM takes a long time to converge as evidenced by the notable long time it took to estimate the smooth functions. Future SI forecasting studies can consider other types of alternative models including the development of deep machine learning models based on the non-parametric QR models introduced in the current study. As a result, apart from non-parametric QR modelling, other non-parametric frameworks, such as the Gaussian process regression, have to be roped in the comparative analysis of SI predictive models to establish a comprehensive forecasting guideline.
